# Current approaches to HIV vaccine development: a narrative review

**DOI:** 10.1002/jia2.25793

**Published:** 2021-11-21

**Authors:** Jiae Kim, Sandhya Vasan, Jerome H. Kim, Julie A. Ake

**Affiliations:** ^1^ US Military HIV Research Program Walter Reed Army Institute of Research Silver Spring Maryland USA; ^2^ Henry M. Jackson Foundation for the Advancement of Military Medicine Bethesda Maryland USA; ^3^ International Vaccine Institute Seoul South Korea

**Keywords:** Adjuvant, HIV, vaccines

## Abstract

**Introduction:**

The development of an effective vaccine to protect against HIV is a longstanding global health need complicated by challenges inherent to HIV biology and to the execution of vaccine efficacy testing in the context of evolving biomedical prevention interventions. This review describes lessons learnt from previous efficacy trials, highlights unanswered questions, and surveys new approaches in vaccine development addressing these gaps.

**Methods:**

We conducted a targeted peer‐reviewed literature search of articles and conference abstracts from 1989 through 2021 for HIV vaccine studies and clinical trials. The US National Library of Medicine's Clinical Trials database was accessed to further identify clinical trials involving HIV vaccines. The content of the review was also informed by the authors’ own experience and engagement with collaborators in HIV vaccine research.

**Discussion:**

The HIV vaccine field has successfully developed multiple vaccine platforms through advanced clinical studies; however, the modest efficacy signal of the RV144 Thai trial remains the only demonstration of HIV vaccine protection in humans. Current vaccine strategies include prime‐boost strategies to improve elicitation of immune correlates derived from RV144, combination mosaic antigens, novel viral vectors, antigens designed to elicit broadly neutralizing antibody, new nucleic acid platforms and potent adjuvants to enhance immunogenicity across multiple classes of emerging vaccine candidates.

**Conclusions:**

HIV vaccine developers have applied lessons learnt from previous successes and failures to innovative vaccine design approaches. These strategies have yielded novel mosaic antigen constructs now in efficacy testing, produced a diverse pipeline of early‐stage immunogens and novel adjuvants, and advanced the field towards a globally effective HIV vaccine.

## 1 INTRODUCTION

Human immunodeficiency virus (HIV) remains a widespread and compelling global health threat with 38 million people living with HIV and 1.7 million new cases in 2019 [1]. Global deployment of antiretroviral therapy and an increasing armamentarium of non‐vaccine HIV prevention tools are being employed to combat the epidemic, but, as discussed elsewhere in this special issue, an effective vaccine will likely be needed to end it [2]. Many technologic advances and research capabilities of the HIV vaccine field have been leveraged rapidly to develop highly successful SARS‐CoV‐2 vaccines; however, among HIV vaccine efficacy trials completed to date, the RV144 Thai trial remains the only study to demonstrate a positive signal with an estimated efficacy of 31% at 3.5 years [3]. We will review current approaches to improve on that result, including vaccine candidates in efficacy trials as well as platforms in earlier stages of clinical development.

## 2 METHODS

We conducted a targeted peer‐reviewed literature search employing PubMed/MEDLINE to access material from 1989 to 2021 and utilized the keywords HIV and/or vaccine, trimer, broadly neutralizing antibody (bNAb), mRNA, adjuvant. The U.S. National Library of Medicine's Clinical Trials database was searched to further identify HIV vaccine clinical trials. The review was informed by the authors’ own experience in the HIV vaccine field and was completed on 18 March 2021.

## 3 DISCUSSION

### 3.1 Challenges to HIV vaccine development

Development of a globally effective HIV vaccine faces manifold challenges. HIV's characteristic sequence diversity poses a formidable obstacle, with circulating strains differing from one another by approximately 20% in relatively conserved proteins and up to 35% in the envelope (Env) [4, 5]. HIV has multiple transmission routes, including intravenous, vaginal or intrarectal sexual transmission, all of which result in widespread systemic viral dissemination within one to two weeks post exposure [6, 7, 8, 9, 10], seeding long‐lived cellular populations [11, 12] and immune‐privileged anatomic reservoir sites [13, 14, 15, 16]. HIV envelope glycans, critical for Env folding and cleavage during maturation [17, 18, 19], also enable HIV to evade the immune system by hiding epitopes [20]. Furthermore, HIV proteins such as Vif and Vpu antagonize host viral restriction factors [21, 22, 23, 24, 25].

In addition to technical obstacles, HIV vaccine efficacy testing is challenged by logistical considerations and advancements in non‐vaccine prevention modalities such as preexposure prophylaxis (PrEP), which must be reflected in the design and conduct of efficacy trials. Ethical considerations for incorporating PrEP into preventive HIV vaccine efficacy trials are further discussed in the commentary by Slack, *et al*. in this issue [26].

However, despite the rapidly evolving virus and prevention landscape, the HIV vaccine field has developed multiple candidates through the successful implementation of efficacy trials (Table 1), demonstrated initial efficacy with RV144, and is now advancing a panoply of new vaccine design approaches into the clinic. These accomplishments highlight the feasibility and promise of a licensed HIV vaccine to end the epidemic.

**Table 1:** Summary of HIV vaccine efficacy trials

**Study**	**Immunogen**	**Adjuvant**	**Schedule**	**Location/study population**	**Date**	**Efficacy**	**Reference**
VAX003 (NCT00006327)	AIDSVAX B/E (subtype B ‐ MN; subtype AE ‐ A244 rgp120)	Aluminium hydroxide	M 0/1/6/12/18/24/30	Thailand/persons who inject drugs	March 1999 to August 2000	No	[27, 28, 31]
VAX004 (NCT00002441)	AIDSVAX B/B (subtype B ‐ MN and GNE8 rgp120)	Aluminium hydroxide	M 0/1/6/12/18/24/30	North America, Netherlands/MSM and high‐isk women	2001 to 2003	No	[29, 30, 31]
STEP HVTN 502	Ad5 expressing subtype B Gag (CAM‐1), Pol (IIIB), Nef (JR‐FL)		M 0/1/2/6	Americas, Australia/MSM and high‐risk heterosexual men and women	December 2004 to March 2007	No	[32, 34, 35]
Phambili HVTN 503 (NCT00413725)	Ad5 expressing subtype B Gag (CAM‐1), Pol (IIIB), Nef (JR‐FL)		M 0/1/2/6	Republic of South Africa/heterosexual men and women	January 2007 to September 2007	No	[33]
RV144 (NCT00223080)	ALVAC‐HIV (vCP1521) expressing Gag and Pro (subtype B LAI), CRF01_AE gp120 (92TH023) linked to transmembrane anchoring portion of gp41 (LAI) AIDSVAX B/E	Aluminium hydroxide	M 0/1/3/6	Thailand/relatively low‐risk men and women	October 2003 to July 2009	Yes ‐ 31%	[36, 37, 38, 39, 40, 41, 42, 43, 44, 45]
HVTN 505 (NCT00865566)	6 DNA plasmids ‐ subtype B Gag, Pol, Nef and subtypes A, B and C Env 4 rAd5 vectors ‐ subtype B Gag/Pol and subtypes A, B and C Env		D 0/28/56/168	USA/Ad5 seronegative MSM	May 2009 to October 2017	No	[46, 47, 48, 49]
HVTN 702 (NCT02968849)	ALVAC‐HIV (vCP2438) expressing Gag and Pro (subtype B LAI), subtype C gp120 (ZM96.C) linked to transmembrane anchoring portion of gp41 (LAI) Bivalent C gp120 (TV1 C/1086 C)	MF59	M 0/1/3/6/12 (18)	Republic of South Africa/heterosexual men and women	October 2016 to (Proj) September 2021	No	
IMBOKODO HVTN 705 (NCT03060629)	Ad26.Mos4.HIV Subtype C gp140	Aluminium phosphate	M 0/3/6/12	RSA, Malawi, Mozambique, Zambia, Zimbabwe/women	November 2017 to (Proj) July 2022		
MOSAICO HVTN 706 (NCT03964415)	Ad26.Mos4.HIV Subtype C gp140 or bivalent gp140 (subtype C/Mosaic)	Aluminium phosphate	M 0/3/6/12	US, Latin America, Italy, Spain/cis‐gender men and transgender individuals who have sex with cis‐gender men and/or transgender Individuals	October 2019 to (Proj) January 2023		
PrEPVacc (NCT04066881)	DNA‐HIV‐PT123 plasmid and AIDSVAX B/E or DNA‐HIV‐PT123 plasmid with trimeric CN54gp140; MVA‐CMDR (Chang Mai double recombinant) and trimeric CN54gp140 Concurrent PrEP administration of either TAF/FTC or TDF/FTC	Aluminium hydroxide or MPLA	M 0/1/6/12	Uganda, Tanzania, Mozambique, Republic of South Africa/men and women	(Proj) January 2020 to (Proj) March 2023		
AMP Study HVTN 703/HPTN 081 (NCT02568215) HVTN 704/HPTN 085 (NCT02716675)	Monoclonal antibody VRC01		M 0/2/4/6/8/10/12/14/16/18	(HVTN 703) Botswana, Kenya, Malawi, Mozambique, Republic of South Africa, Tanzania, Zimbabwe/women (HVTN 704) US, Brazil, Peru, Switzerland/men	(HVTN 703) April 2016 to December 2020 (HVTN 704) May 2016 to March 2021	No	[76]

Abbreviations: Proj, projected; MSM; men who have sex with men; RSA, Republic of South Africa; US, United States.

### 3.2 Early vaccine efficacy trials

#### 3.2.1 VAX003 and VAX004

The first Phase 3 HIV vaccine efficacy trials were VAX003 and VAX004, which both utilized a bivalent protein formulation with alum adjuvant. VAX003 participants were persons who inject drugs in Thailand and utilized bivalent subtype B and AE proteins [27, 28], and VAX004 enrolled men who have sex with men (MSM) and women at risk for heterosexual acquisition of HIV in the Americas with bivalent subtype B proteins [29, 30]. In both trials, the vaccines did not prevent HIV infection, and there was no significant effect on viral load, CD4^+^ T‐cell count, or disease progression [31], suggesting that bivalent protein vaccination alone is insufficient to induce protective efficacy.

#### 3.2.2 STEP (HVTN 502) and Phambili (HVTN 503) trials

Results of the VAX003 and VAX004 trials prompted a shift to strategies for eliciting HIV‐specific T‐cell responses with vector‐based vaccines. The STEP trial evaluating a replication defective adenovirus serotype 5 (Ad5) vectored vaccine enrolled MSM, sex workers, and participants with elevated heterosexual risk in the Americas and Australia [32]. The trial was halted by the Data Safety Monitoring Board (DSMB) after initial data showed an increase in HIV infection among uncircumcised males and/or Ad5 seropositive vaccinees. Interim analysis revealed an infection rate in per protocol male vaccinees double that of placebo recipients. The Phambili trial, which enrolled participants in the Republic of South Africa (RSA) [33] was terminated early based on the results of the STEP trial, and preliminary data revealed a higher incidence of HIV infection in the vaccinated group compared to placebo, without impact on viral load or disease progression. *In vitro* experiments demonstrated that Ad5‐specific CD4^+^ T cells are highly susceptible to HIV infection [34], and that these cells are preferentially lost in HIV‐1‐positive individuals [35]. These studies raised important questions about pre‐existing anti‐vector immunity and concern about the use of Ad5 vectored vaccines where Ad5 is prevalent.

#### 3.2.3 RV144 Thai trial

RV144, performed by the United States Army and the Thai Ministry of Public Health with support from the National Institutes of Health (NIH), was conducted from 2003 to 2009 in Rayong and Chon Buri provinces and enrolled over 16,000 participants at relatively low heterosexual risk for HIV infection. This was the first efficacy trial employing a pox‐protein prime‐boost strategy: ALVAC‐HIV (vCP1521), a recombinant canarypox vector, was boosted with the bivalent protein AIDSVAX B/E adsorbed to aluminium hydroxide aiming to elicit both cellular and humoral responses. The regimen showed 60% efficacy at 12 months post immunization; however, this waned to 31% at 3.5 years [36]. Despite the modest result, RV144 rekindled hope in the feasibility of an effective HIV vaccine and raised the central question of improving durability of protection.

Extensive analyses enabled identification of immune correlates of risk from RV144 (Table 2) [37, 38, 39, 40, 41, 42, 43, 44, 45]. Importantly, the vaccine did not elicit neutralizing antibodies, opening the door to investigate non‐neutralizing mechanisms of protection. IgG antibody binding to the V1V2 region of envelope correlated inversely with the rate of HIV acquisition, and binding of plasma IgA antibodies to envelope correlated directly with the rate of infection. The correlates analysis also indicated that avidity of IgG for envelope, antibody‐dependent cellular cytotoxicity (ADCC) and phagocytosis (ADCP), and Env‐specific CD4^+^ T cells were inversely correlated with risk of infection. Subsequent sieve analysis revealed that the vaccine caused selective effects on the V2 region in breakthrough viruses [45].

**Table 2:** Correlates of HIV protection determined in RV144

		**Reference**
Antibody binding to Env	IgG to V2	[37, 38]
	IgG to V2 and V3	[39]
	IgG3 to V1V2	[40]
	Avidity of IgG for Env	[37]
	Low IgA	[41]
Antibody functions	IgG mediated ADCC	[42]
	IgG3 associated ADCC and ADCP	[43]
	Lower IgA enables ADCC function	[41]
Cellular	Polyfunctional Env‐specific CD4^+^ T cells	[44]

#### 3.2.4 HVTN 505 trial

The next efficacy results reported were those of HVTN 505, which was designed to answer questions about Ad5 vectors left outstanding by the STEP/Phambili results. It was conducted in the United States in Ad5 sero‐negative men and transgender females who have sex with men to test a DNA/rAd5 prime‐boost regimen, inclusive of Env, Gag, Pol and Nef immunogens [46]. Of note, the V1V2 regions were deleted from the subtype B Env gene in this formulation. The DSMB halted the trial after the first interim analysis revealed that the vaccine did not prevent infection nor reduce HIV viral load [47]. *Post hoc* analysis of HVTN 505 revealed that ADCP, binding to FcγRIIa, and HIV‐1 Env IgG3 correlated to reduced acquisition risk of HIV [48]. Additional work revealed that certain polymorphisms of FcγR modulate HIV acquisition after vaccination, demonstrating the potential for host genetics to impact vaccine efficacy [49].

### 3.3 Follow‐up trials building on RV144

#### 3.3.1 RV305, RV306 and RV328 trials

With the observation of waning immunity and time‐dependent efficacy in RV144 [3, 36], two early‐phase trials examined strategies to improve durability of responses by boosting with vaccine regimen components. RV305 enrolled vaccinated individuals without HIV from RV144 after six to eight years, evaluating two immunizations of ALVAC‐HIV, AIDSVAX B/E, or both compared to placebo. ALVAC‐HIV boosting alone did not induce significant titers against any of the HIV antigens examined. Those who received ALVAC‐HIV plus AIDSVAX B/E or AIDSVAX B/E alone had IgG responses against gp120 and gp70‐V1V2 at levels significantly higher than peak immunogenicity in RV144, indicating that the priming RV144 vaccination series six to eight years earlier had evoked memory responses [50]. Interestingly, the magnitude of those increases was higher after the initial late boost at month 0 than after the second late boost at month 6, suggesting that the duration of the boosting interval influences responses. Repeated boosting induced antibodies with qualities of bNAbs such as greater somatic hypermutation and longer immunoglobulin heavy‐chain complementarity‐determining region 3 (HCDR3) length [51]. However, an additional delayed boost skewed responses towards the IgG4 subclass, associated with reduced non‐neutralizing function (unpublished data). Therefore, boosting repeatedly with the same antigen may fall short in achieving durably functional immune responses. This trial also confirmed that similar HIV‐specific antibody responses were present in cervicovaginal and rectal secretions and semen, which, in RV144, may have impacted early viral pathogenesis, resulting in prevention of infection or interruption of critical steps early in acquisition [52].

RV306 recapitulated the RV144 regimen and added boost immunizations with either AIDSVAX B/E (12 months) or ALVAC‐HIV/AIDSVAX B/E (12, 15 or 18 months) to determine optimal boost timing. The impact of late boosting was demonstrated by lower plasma IgG responses at 24 months in those receiving the boost at 12 months compared to those receiving the immunizations at 15 and 18 months. Delayed boosting also increased CD4^+^ T‐cell functionality and polyfunctionality [53].

#### 3.3.2 HVTN 097 and HVTN 100 trials

Following the promising results from RV144, HVTN 097 was conducted in RSA employing the RV144 vaccine formulation and schedule. The study demonstrated overall response rates of plasma IgG and Env‐specific CD4^+^ T cells expressing IFN‐γ and/or IL‐2 similar to those elicited in RV144 [54, 55].

HVTN 100 was conducted in RSA with a pox‐protein regimen re‐tooled for the South African subtype C epidemic: the ALVAC‐HIV vCP2438 expressed HIV subtype C gp120, subtype B gp41, gag and protease followed by a bivalent subtype C (TV1/1086) gp120 boost. Also, an alternative adjuvant, the MF59 oil‐in‐water emulsion, was substituted for the aluminium hydroxide used in RV144. This vaccine regimen had a similar schedule to RV144, with an additional boost at month 12, and was shown to be safe and well tolerated. All vaccine recipients developed gp120 binding antibodies, which were significantly increased compared to RV144. The regimen also induced higher CD4^+^ T‐cell responses to the corresponding envelope protein. Although IgG antibody responses directed at 1086_V1V2 were lower in HVTN 100 relative to those elicited by the RV144 regimen in HVTN 097 (70.5% vs. 99%), responses exceeded the predicted threshold needed for 50% vaccine efficacy, and the results from this trial met the go‐no‐go criteria for advancing to the HVTN 702 trial [56].

#### 3.3.3 HVTN 702 Uhambo efficacy trial

Uhambo, the HVTN 702 Phase 2b/3 study in RSA, began in 2016 and enrolled participants at risk for HIV. These participants were sexually active men and women aged 18 to 35 and were offered the local HIV‐prevention standard of care, including access to PrEP. In February 2020, interim analysis results revealed that there was no significant evidence of decreased or increased infection rates associated with the vaccine regimen. The analysis was performed with 2694 vaccinees and 2689 placebo recipients, with 129 HIV infections among the vaccinees and 123 HIV infections among the placebo recipients. Given this lack of efficacy, The National Institute of Allergy and Infectious Diseases (NIAID) halted the trial at the recommendation of the DSMB.

HVTN 702 was highly successful operationally; however, the results indicating the vaccine failed to reproduce or amplify the efficacy of RV144 were disappointing. To understand this outcome, it is useful to examine the differences between the two studies (Table 3).

**Table 3:** Summary of RV144 and HVTN 702 efficacy trials

		**RV144**	**HVTN 702**
Viral	Viral subtype	AE	C
	Viral diversity	Relatively homogenous	Highly diverse
Population	HIV risk/incidence	0.28%	Approximately 4%
	Host genetics	Thai	African
Vaccine	Adjuvant	Aluminium hydroxide	MF59
	ALVAC inserts	Subtype AE vCP1521	Subtype C vCP2438
	Protein boost	Bivalent AE/B (A244/MN)	Bivalent C (TV1 C/1086 C)
	Protein dose	Higher (300 μg of each protein)	Lower (100 μg of each protein)
	Dosing schedule	M 0/1/3/6	M 0/1/3/6/12 (18)

First, the targeted viruses and populations were both very different. RV144 was conducted in a CRF01_AE predominant region, and HVTN 702 was conducted where subtype C virus circulates. Also, the population studied in Thailand was at dramatically lower risk for infection, as the HIV incidence was almost 15‐fold lower in Thailand (0.28%) compared to RSA (approximately 4%). Differences in host genetics of the populations participating in the clinical trials may have played a role [57]. Critically, the vaccine components also differed substantially between the regimens: both the ALVAC inserts and the protein boosts differed. The amount of bivalent protein administered also varied, with a dose of 600 μg in RV144 and 200 μg in HVTN 702. The lower dose of the bivalent gp120 was administered due to the use of MF59, which contrasted with the aluminium hydroxide used in RV144.

In considering the discrepant outcomes of these studies, the critical question to be answered is ‐ what provides protection? For example, the implications of using a different adjuvant may be significant: a non‐human primate (NHP) study examined MF59 versus aluminium hydroxide. The study standardized the baseline risk of infection, challenge virus and other vaccine components. Protection was observed only among animals receiving aluminium hydroxide – containing immunizations. Although higher plasma antibody titers were observed with MF59, they did not protect from acquisition, and mucosal antibodies were the outcome that correlated with protection [58]. This study underscores that higher magnitude of immune responses does not necessarily correspond to better protection and that the quality and location of the response is important. Also, genes expressed after vaccination may play a role in protection. The RV144 vaccine regimen strongly induced the IFNγ pathway, more specifically the activation of IRF7, which was associated with reduced risk of acquisition of infection [59].

Although HVTN 702 was unsuccessful protecting against HIV acquisition, investigators can further examine factors that may have impacted infection within the study population. To inform subsequent progress in HIV vaccine development, it is critical to understand correlates of risk from HVTN 702 across immune and anatomic compartments. These findings can then provide insight for approaching the current diversity of strategies being undertaken to achieve a successful HIV vaccine (Table 4).

**Table 4:** Summary of early‐phase HIV vaccine trials

**Study**	**Phase**	**Immunogen**	**Adjuvant**	**Schedule**	**Location/study population**	**Date**	**Reference**
HVTN 097 (NCT02109354)	1	ALVAC‐HIV (vCP1521) expressing Gag and Pro (subtype B LAI), CRF01_AE gp120 (92TH023) linked to transmembrane anchoring portion of gp41 (LAI) AIDSVAX B/E	Aluminium hydroxide	M 0/1/3/6	RSA/heterosexual men and women	June 2013 to February 2015	[54, 55]
HVTN 100 (NCT02404311)	1, 2	ALVAC‐HIV (vCP2438) expressing Gag and Pro (subtype B LAI), subtype C gp120 (ZM96.C) linked to transmembrane anchoring portion of gp41 (LAI) Bivalent C gp120 (TV1 C/1086 C)	MF59	M 0/1/3/6/12 (18)	RSA/heterosexual men and women	February 2015 to August 2018	[56]
HVTN 111 (NCT02997969)	1	DNA‐HIV‐PT123: DNA plasmid encoding subtype C Gag (ZM96), Env (ZM96), and Pol‐Nef (CN54) Bivalent C gp120 (TV1/1086)	MF59	M 0/1/3/6	RSA, Tanzania, Zambia/men and women	June 2016 to July 2018	[60]
APPROACH HIV‐V‐A‐004 IPCAVD 009 (NCT02315703)	1, 2	Ad26.Mos.HIV vector followed by boosts of Ad26.Mos.HIV and gp140 (high or low dose); Ad26.Mos.HIV alone; MVA‐mosaic and gp140 (high or low dose); MVA‐mosaic alone; or gp140	Aluminium phosphate	M 0/1/3/6/12	USA, Rwanda, RSA, Thailand, Uganda/men and women	December 2014 to (Proj) July 2022	[65]
TRAVERSE HVTN 117 (NCT02788045)	1, 2a	Ad26.Mos.HIV or Ad26Mos4.HIV Subtype C gp140	Aluminium phosphate	M 0/3/6/12	USA, Rwanda/men and women	July 2016 to (Proj) April 2023	[66]
ASCENT HVTN 118 (NCT02935686)	1, 2a	Ad26.Mos4.HIV Subtype C gp140 or bivalent gp140 (subtype C/mosaic)	Aluminium phosphate	M 0/3/6/12	USA, Kenya, Rwanda/men and women	March 2017 to (Proj) May 2023	[67]
HIVCORE 005/6 (NCT04553016)	1	ChAdOx1.tHIVconsv1 MVA.tHIVconsv3 and MVA.tHIVconsv4		M 0/1	Kenya, Uganda, Zambia/men and women	(Proj) March 2021 to (Proj) June 2022	
IAVI W001 (NCT03699241)	1	BG505 SOSIP.664 gp140	AS01B		USA, Kenya/men and women	December 2018 to (Proj) May 2020	
ACTHIVE‐001 (NCT03961438)	1	ConM SOSIP.v7 gp140	MPLA	M 0/2/6	Netherlands/men and women	November 2019 to (Proj) November 2022	
EAVI2020_01 (NCT03816137)	1	ConM SOSIP, EDC ConM SOSIP, ConS UFO, EDC ConS UFO and mosaic SOSIPs	MPLA	M 0/3/6/12	UK/men and women	March 2019 to (Proj) December 2021	
IAVI G001 (NCT03547245)	1	eOD‐GT8 (self‐assembling nanoparticle with HIV Env)	AS01B		USA/men and women	June 2018 to (Proj) December 2020	
IAVI C101 (NCT04224701)	1	BG505 SOSIP GT1.1 gp140	Present (from GSK)	M 0/2/6	USA, Netherlands/men and women	August 2020 to (Proj) July 2022	
IAVI study (NCT01264445)	1	Ad35 expressing Gag, RT, Int, and Nef; fusion protein expressing subtype B p24, RT, Nef and p17	AS01	M 0/1/4	Kenya, Uganda, Zambia/men and women	February 2011 to November 2012	[10]
RV546 (NCT04658667)	1	Full‐length single‐chain (FLSC) gp120‐CD4 chimera subunit HIV‐1 vaccine (IHV01), A244 gp120	Aluminium hydroxide ALFQ	D 0	Thailand/RV306 participants	(Proj) August 2021 to (Proj) October 2022	
HVTN 137 (NCT04177355)	1	BG505 SOSIP.664 gp140	3M‐052‐AF CpG 1018 GLA‐LSQ Aluminium hydroxide	M 0/2/(6)	USA/men and women	January 2020 to (Proj) May 2022	

Abbreviations: Proj, projected; RSA, Republic of South Africa; USA, United States of America.

#### 3.3.4 HVTN 111 trial

While HVTN 100 (and subsequently HVTN 702) adopted a pox‐protein approach to parallel RV144, HVTN 111 adopted a DNA priming approach, employing a subtype C DNA prime (DNA‐HIV‐PT123) with the same bivalent subtype C gp120 boost used in the HVTN 100 regimen. Evaluating strategies for enhancing RV144 correlates of protection, Moodie *et al*. examined antibody and cellular responses of both HVTN 100 and HVTN 111 [60]. Although both the ALVAC and DNA priming regimens induced high binding and neutralizing titers, the DNA prime induced significantly higher CD4^+^ T‐cell response rates compared to the ALVAC prime. Interestingly, the vaccinees from HVTN 111 had higher magnitude of binding antibodies to the V1V2 region and had higher neutralizing responses to tier‐1 viruses compared to the HVTN 100 vaccinees. This study highlighted the potential for DNA priming to enhance the magnitude of elicited immune responses in protein‐boosted HIV vaccine regimens.

### 3.4 New approaches for an HIV vaccine

#### 3.4.1 Ad26 mosaic trials

The Ad26 mosaic vaccine program developed by Janssen Pharmaceuticals has combined viral vectors (adenovirus serotype 26 or modified vaccinia Ankara ‐ MVA), protein boosts and immunogen sequences optimized to address global HIV diversity in the creation of polyvalent “mosaic” antigens. Fischer *et al*. explained the approach for designing antigens that focus on eliciting T‐lymphocyte responses as well as neutralizing antibodies by including Env as part of the design [61]. The mosaic antigens are generated from natural sequences, which include common potential epitopes, while excluding rare sequences.

The clinical use of these mosaic antigens was informed by a series of NHP studies [62, 63, 64], and the initial regimen selection clinical trial for this platform was the APPROACH study. This Phase 1/2 trial assessed seven regimens containing Ad26 or MVA vectors expressing mosaic antigens, with some groups boosted with high or low doses of gp140 adsorbed to aluminium phosphate. All regimens were safe and well tolerated [65], and binding antibody responses to autologous clade C gp140 was detected in all groups following the second prime immunization at month 3. After the first boost immunization at month 6, most groups maintained 100% antibody response. The groups that received the gp140 boost had the highest ELISA antibody titers, the magnitude of which was dose‐dependent. In comparison to the gp140‐only group, the inclusion of either Ad26.Mos.HIV or MVA‐mosaic increased responses. The utility of the mosaic antigens was demonstrated by the ability to elicit binding IgG responses to cross‐clade transmitted/founder Envs, Envs isolated from chronically infected individuals, and to consensus sequence Envs that were similar to the vaccine homologous responses [65]. Similar to RV144 vaccinees, a substantial number of subjects showed responses to gp70‐V1V2 antigens and the IgG subclasses elicited were IgG1 and IgG3. The highest ADCP responses were observed in the gp140‐boosted groups, particularly those primed with a viral vector. Serum neutralizing activity was only detected against tier‐1HIV variants [65].

In parallel with the APPROACH study, an NHP study (13‐19) was conducted and observed similar immunogenicity. All animals underwent six intrarectal challenges with SHIV‐SF162P3, with the Ad26.Mos.HIV/gp140 (aluminium phosphate) group demonstrating the most favourable results: eight out of twelve animals were protected from the entire series of challenges, demonstrating a 94% reduction in per acquisition risk and 66% complete protection [65].

Four more clinical trials have built on these initial findings: TRAVERSE, IMBOKODO, ASCENT, and MOSAICO. TRAVERSE is a Phase 1/2a study that assessed the safety and immunogenicity of a trivalent Ad26.Mos.HIV compared to a tetravalent Ad26.Mos4.HIV prime in the context of a subtype C gp140 boost adjuvanted with aluminium phosphate. All vaccinees developed gp140 binding IgG antibodies after two immunizations of either the trivalent or tetravalent formulations; however, the tetravalent formulation elicited higher total IgG geometric mean titers. ADCP function and Env‐specific CD4^+^ T‐cell response rates were also improved with the tetravalent vaccine [65], and the Ad26.Mos4.HIV formulation has advanced into the IMBOKODO proof‐of‐concept efficacy trial. This Phase 2b study conducted in southern Africa has completed enrollment with women at risk for HIV, and the primary completion date is projected for 2022.

ASCENT is a Phase 1/2a study that assessed the Ad26.Mos4.HIV prime with either a monovalent subtype C gp140 boost or a bivalent combination of subtype C gp140 and mosaic gp140. Both regimen boosts were adjuvanted with aluminium phosphate and showed high Env‐specific binding antibody levels with similar subclass distribution. Clade C responses were not attenuated by the half dose administered in the group containing the mosaic gp140 boost, which also demonstrated improved subtype B responses. CD4^+^, but not CD8^+^, T‐cell responses were increased in the bivalent regimen compared to the subtype C gp140 alone [67]. This Ad26 mosaic vaccine regimen with bivalent protein boosting has subsequently advanced into the MOSAICO Phase 3 licensure trial in the Americas and Europe, which began in October 2019 and plans an estimated enrollment of 3800 cis‐gender men and transgender individuals who have sex with cis‐gender men and/or transgender individuals. If efficacy is seen in IMBOKODO and/or MOSAICO, those results will add to the modest success of RV144 to demonstrate the ability of non‐neutralizing antibody and cellular mechanisms to afford protection and expand our knowledge of correlates of protection to facilitate bridging studies to additional populations and geographic regions.

Geographic and participant diversity as well as broad collaboration have been strengths of the Janssen clinical programme, which has engaged multiple HIV vaccine research institutions, networks and laboratories since its inception. The mosaic concept is being put to test with efficacy efforts across multiple HIV subtypes as well as a diversity of participant gender, demographic and risk factors with appropriate engagement of these communities, leveraging established capabilities of the HVTN and other partners. All these considerations have implications not just for demonstrating and understanding vaccine protection, but also for the eventual deployment of vaccines to communities at risk.

#### 3.4.2 PrEPVacc trial

Another highly collaborative effort, PrEPVacc, is an African‐led vaccine efficacy trial supported by the European and Developing Countries Clinical Trials Partnership (EDCTP) incorporating a PrEP comparison into a study design evaluating two vaccine candidates. The first vaccine regimen, previously evaluated in HVTN105 (NCT02207920), is a DNA‐HIV‐PT123 plasmid prime (as in HVTN111) plus an AIDSVAX B/E boost. The second vaccine regimen is a DNA‐HIV‐PT123 with CN54gp140 prime followed by an MVA‐CMDR (Chang Mai double recombinant) and CN54gp140 boost. The two vaccine regimens will be compared against placebo, and participants are offered PrEP with TDF/FTC or TAF/FTC during the period of the first three immunizations. PrEPVacc utilizes a multiarm, multistage adaptive trial design that enables the efficient evaluation of multiple vaccine regimens and also employs an averted infections ratio methodology to determine efficacy. Vaccinations have commenced and the study is estimated to be completed in 2023.

#### 3.4.3 New viral vectors

Given the insufficient efficacy seen with Ad5 and ALVAC vaccines, other viral vectors are being explored for improved HIV vaccine regimens. Strategies employing Ad26 or Ad35 vectors have been shown to elicit robust humoral and cellular immune responses, and importantly, pre‐existing anti‐vector antibodies do not affect vaccine safety or immunogenicity [68, 69, 70, 71]. Ad4 is a live virus vaccine vector under clinical evaluation. NCT01989533 is examining an Ad4‐HIV vaccine regimen consisting of an Ad4‐mosaic Gag prime and an Ad4‐EnvC150 boost. The vaccine is formulated as enteric‐coated capsules to be delivered orally or as an aqueous formulation for intranasal administration.

The human cytomegalovirus (CMV) vector is another promising novel vectored approach for HIV vaccines. NHPs vaccinated with CMV/SIV vectors induced persistent and high‐frequency SIV‐specific memory T‐cell responses at potential sites of SIV replication. These responses were associated with durable aviremic control of SIV infection in 50% of the NHPs, and this protection correlated with CD8^+^ T‐cell responses [72, 73]. The first clinical trial to examine the safety and immunogenicity of a CMV vector‐based vaccine, VIR‐1111, is currently recruiting healthy CMV seropositive participants.

HIVCORE 005/6 is a Phase 1 EDCTP clinical trial testing three experimental vaccines with other viral vectors: mosaic immunogens delivered by a prime‐boost regimen containing a chimpanzee adenovirus vector (ChAdOx1.tHIVconsv1) and a dual boost of a nonreplicating MVA vector (MVA.tHIVconsv3 and MVA.tHIVconsv4). The ChAdOx1 vector is a component of the Astra Zeneca COVID‐19 vaccine, which has been granted a conditional marketing authorization or emergency use in over 50 countries [74, 75].

#### 3.4.4 Antibody mediated protection (AMP) trials

The antibody mediated protection (AMP) trials evaluated the protective efficacy of passive immunization with either low (10 mg/kg) or high (30 mg/kg) doses of the bNAb VRC01 delivered every eight weeks in participants at increased risk for HIV acquisition in sub‐Saharan Africa (HVTN 703/HPTN 081) and the Americas (HVTN 704/HPTN 081) [76]. The studies did not demonstrate overall efficacy; however, as pioneered in earlier vaccine efficacy trials, evaluation of breakthrough viruses yielded critical insight. The bNAb was 75% effective against VRC01‐sensitive isolates (those with 80% inhibitory concentration less than 1 μg/ml) over the course of the study, demonstrating proof of concept for bNAb prophylaxis. However, only a minority of viruses (30% in the placebo groups) were VRC01‐sensitive, highlighting the need for broader and more potent bNAbs, likely in combination targeting multiple HIV epitopes. The results also have implications for active vaccination strategies to elicit bNAb: that bar is high, and to achieve protection through purely neutralizing mechanisms, active vaccines will likely need to target multiple HIV epitopes as well.

#### 3.4.5 Native‐like envelope trimers

To actively induce bNAb responses, several groups have been advancing novel envelope immunogens. Native‐like envelope trimers are designed to present multiple bNAb epitopes mimicking how these epitopes appear on the Env spike, with the BG505 SOSIP.664 construct emerging as the prototypic native‐like soluble trimer [77]. Compared to earlier HIV protein immunogens, these trimers are purer and more thermally stable. The approach to the design of these HIV immunogens is further detailed by Sanders and Derking [78].

Several clinical trials are underway evaluating SOSIP trimers. W001 is a Phase 1 IAVI clinical trial testing the BG505 SOSIP.664 gp140 at various doses in combination with the AS01B adjuvant. The Phase 1 ACTHIVE‐001 trial is examining the ConM SOSIP.v7 gp140 adjuvanted with monophosphoryl lipid A (MPLA) liposomes (ConM is a consensus of the consensus sequences of each clade in group M). Lastly, the EAVI2020_01 Phase 1 trial is evaluating prime‐boost combinations with various forms of SOSIP gp140s including the ConM SOSIP, EDC ConM SOSIP, ConS UFO, EDC ConS UFO, and mosaic SOSIPs.

#### 3.4.6 Serial bNAb germline targeting

Designing immunogens to engage bNAb precursors and their intermediates through sequential immunization [79, 80] is an alternate approach to bNAb elicitation, as further described by Williams *et al*. [81]. At HIVR4P 2021, encouraging results of the IAVI Phase 1 G001 trial were announced. This study demonstrated that the eOD‐GT8 60mer, a self‐assembling nanoparticle comprised of HIV Env, adjuvanted with AS01B, elicited detectable VRC01‐class IgG B cells in 97% of vaccinees showing initial proof of concept in humans for the germline targeting approach. Another vaccine candidate designed to induce bNAbs, adjuvanted BG505 SOSIP.GT1.1 gp140, is being examined at two different doses in the IAVI C101 Phase 1 clinical trial.

#### 3.4.7 Messenger RNA vaccines

With many of its roots in vaccine development for cancer, HIV and other infectious diseases, messenger RNA (mRNA) is gaining momentum as a platform following the striking success of SARS‐CoV‐2 mRNA vaccines. mRNA uses host cells to manufacture protein immunogens that can elicit potent antibody and cellular responses [82]. The platform has the potential for cost‐effective and scalable GMP manufacturing, without concern for genome integration or the formation of infectious particles [83, 84].

As of July 2021, two mRNA vaccines have shown over 90% efficacy and received Emergency Use Authorization for the prevention of COVID‐19 [85, 86]. The success of these products leveraged lessons in mRNA delivery and immunogen stabilization that have been learnt in the HIV context [82, 87, 88] and are now being applied by multiple groups in the development of new HIV vaccines. For example, a m1ψ‐mRNA‐LNP construct encoding a variant of clade B HIV R3A Env elicited potent immune responses in mice, and a single 50 μg immunization of mRNA‐LNP in NHPs resulted in a significant increase in Tfh cell frequency in the draining lymph nodes compared to those that received double‐stranded RNA‐adjuvanted Env protein immunization. The mRNA‐LNP immunization elicited potent antibody neutralization activity against a highly sensitive clade C virus. These neutralization antibody titers were stable through 12 months post vaccination [89].

Moyo *et al*. explored the potential of a self‐amplifying mRNA (saRNA) encoding six highly conserved regions of the HIV genome (Gag, Pol). A single immunization with this RNA formulation induced specific CD4^+^ and CD8^+^ T cells in BALB/c mice, which were well maintained 22 weeks after vaccination [90]. The immunization also induced CD4^+^ and CD8^+^ memory subtypes. These early results demonstrate promise for the elicitation of durable immune responses with mRNA‐based vaccine formulations against HIV.

#### 3.4.8 New adjuvants

The decrease in efficacy from 60% at 12 months post immunization to 31% at 3.5 years in RV144 and rapid decline in humoral immune responses to many gp120 antigens, highlights the requirement for, increasing durability as well as magnitude of protection [3, 36]. New adjuvants have the potential to augment protein vaccine immune responses to achieve this goal.

Four types of adjuvants are currently included in licensed vaccine formulations: aluminium salts [91], liposomes or virosomes [92], oil‐in‐water emulsions [93] and aluminium salt containing adsorbed MPLA [94]. The AS01 adjuvant system, which includes liposomes containing MPLA and a saponin from the *Quillaja saponaria* tree bark, QS21 [95, 96], has been utilized in vaccines for multiple pathogens [97, 98, 99], and the AS01B formulation is employed in the highly effective Shingrix herpes zoster vaccine [98]. In a human clinical trial study examining an HIV vaccine regimen containing protein immunizations of gp120/Nef Tat with AS01B, the formulation induced strong and durable CD4^+^ and CD8^+^ T‐cell responses with multifunctional profiles [100]. More recently, an IAVI clinical trial employed AS01B in an HIV prime‐boost vaccine strategy evaluating an Ad35 vectored vaccine (expressing Gag, reverse transcriptase, integrase and Nef) with a fusion protein (expressing clade B p24, RT, Nef and p17) adjuvanted with AS01B. This regimen was well tolerated with durable CD4^+^ and CD8^+^ responses [101].

The Walter Reed Army Institute of Research has developed the Army Liposome Formulation (ALF) family of liposomal adjuvants containing MPLA. These formulations can contain aluminium hydroxide gel (ALFA), QS21 (ALFQ) or both (ALFQA). ALF adjuvants are nontoxic and have been employed in vaccine formulations against several diseases including HIV, malaria, and campylobacteriosis [102, 103, 104, 105, 106]. Supported by favourable NHP immunogenicity [107], a Phase 1 clinical trial (NCT04268420) of the FMP013 circumsporozoite protein malaria vaccine with ALFQ has begun and safely dose‐escalated [108].

In the HIV space, a regimen of live‐attenuated MVA encoding HIV‐1 Gag, Pol and Env, with a multimeric gp145 protein boost adjuvanted with ALFA resulted in NHP protection from multiple challenges of SHIV‐1157ipd3N4 [109]. The RV546 Phase 1 trial will assess a full‐length single chain gp120‐CD4 chimera subunit HIV‐1 vaccine with alum (IHV01) paired with A244 gp120, in the absence or presence of ALFQ. This study will enroll RV306 participants, harnessing extant memory responses and evaluating antibody response durability and breadth of antibodies directed to the CD4‐induced (CD4‐i) epitope that preferentially binds to the CD4‐gp120 complex [110].

The HVTN 137 Phase 1 trial is currently underway evaluating the safety and immunogenicity of BG505 SOSIP.664 with several adjuvants and the imidazoquinolinone TLR‐7/8 agonist 3M‐052. Part A of the trial dose escalates 3M‐052 and alum with gp140, and Part B will utilize the highest tolerated dose of 3M‐052 with alum. Part B will also examine other adjuvants with gp140 including CpG with alum, GLA‐LSQ (GLA and QS‐21) and alum alone. Lessons from exploration of novel adjuvants may yield cross‐cutting progress across immunogen platforms targeting both neutralizing and non‐neutralizing antibody mechanisms of protection.

## 4 CONCLUSIONS

Although effective HIV treatment and prevention options are increasingly available, due to limited accessibility and/or other treatment challenges, the urgent need for an HIV vaccine remains. Competing strategies emphasize particular immunologic approaches; however, the exceptional diversity and resilience of HIV will likely necessitate a multiplicity of reinforcing immunologic mechanisms for success. As vaccine technologies and clinical trial networks developed to combat HIV were leveraged in the SARS‐CoV‐2 response, the unprecedented collaboration, transparency, industry engagement and rapid clinical translation with progression to parallel efficacy testing for multiple vaccine concepts that characterized the coronavirus response can propel HIV vaccine development. Important efficacy trials are underway and innovative HIV vaccine design approaches have yielded a robust and diverse pipeline of early‐stage candidates with the potential to incorporate next‐generation adjuvants. The stage is set for HIV vaccine developers to move these concepts forward together to achieve the ultimate public health tool of an effective HIV vaccine.

## COMPETING INTERESTS

The authors declare that they have no competing interests.

## AUTHORS’ CONTRIBUTIONS

JK drafted the initial version of the manuscript. SV, JHK and JAA reviewed and revised the manuscript. All authors approved the final version of the manuscript.

## FUNDING

This work was supported by a cooperative agreement (W81XWH‐18‐2‐0040) between the Henry M. Jackson Foundation for the Advancement of Military Medicine, Inc., and the U.S. Department of Defense (DOD).

## DISCLAIMER

The views expressed are those of the authors and should not be construed to represent the positions of the U.S. Army, the Department of Defense, or HJF.
